# Recent Patents of Interest to Dentists

**Published:** 1907-10

**Authors:** 


### PATENTS.

### RECENT PATENTS OF INTEREST TO DENTISTS.

864054. Tooth Brush, Albert Abrams, San Francisco, Cal.
863896. Receptacle for Toothpicks, Richard Beckmann, Charlottenburg, Germany.
864465. Dental Filling, John Hood, Boston, Mass.
864569. Dental Plugger, George H. Shannon, Cambridge, N. Y.
865112. Contouring Gold Tooth Crowns, Francis LaChapelle, San Francisco, Cal.
865823. Apparatus for the Making of Molds for the Casting of Dental Fillings and the Like, Wm. H. Taggart, Chicago, Ill.
865733. Dental Clamp, Henry B. Weagant, Owen Sound, Ont., Can.
866176. Artificial Denture, Francis Ainsworth, St. Johns, N. B. Can.

866559. Dental Plate Punch, Edward E. Bartram, Los Angeles, Cal.
866518. Drilling Apparatus for Dentists, Paul Repsold, Riga, Russia.
866304. Removable Artificial Denture, Finnis E. Roach, Chicago, Ill.
866305. Fusible Porcelain Cement and Strengthening Backing for Artificial Dentures, Finnis E. Roach, Chicago, Ill.

Copies of above patents may be obtained for fifteen cents each, by addressing John A. Saul, Solicitor of Patents, Fendall Building, Washington, D. C.

